# A global dataset of nitrogen fixation rates across inland and coastal waters

**DOI:** 10.1002/lol2.10459

**Published:** 2025-06-01

**Authors:** Robinson W. Fulweiler, Megan E. Berberich, Shelby A. Rinehart, Jason M. Taylor, Michelle C. Kelly, Nicholas E. Ray, Autumn Oczkowski, Sawyer J. Balint, Alexandra H. Geisser, Catherine R. Mahoney, Mar Benavides, Matthew J. Church, Brianna Loeks, Silvia E. Newell, Malin Olofsson, Jimmy C. Oppong, Sarah S. Roley, Carmella Vizza, Samuel T. Wilson, Peter M. Groffman, J. Thad Scott, Amy M. Marcarelli

**Affiliations:** 1 Department of Earth and Environment, Boston University, Boston, Massachusetts, USA;; 2 Department of Biology, Boston University, Boston, Massachusetts, USA;; 3 Department of Biological Sciences, Michigan Technological University, Houghton, Michigan, USA;; 4 Department of Biodiversity, Earth and Environmental Science, Drexel University, Philadelphia, Pennsylvania, USA;; 5 Water Quality & Ecology Research Unit, United States Department of Agriculture, Agricultural Research Service, National Sedimentation Laboratory, Oxford, Mississippi, USA;; 6 School of Marine Science & Policy, University of Delaware, Lewes, Delaware, USA;; 7 Atlantic Coastal Environmental Sciences Division, United States Environmental Protection Agency, Narragansett, Rhode Island, USA;; 8 National Oceanography Centre, European Way, Southampton, UK;; 9 Aix Marseille Univ, Université de Toulon, CNRS, IRD, Marseille, France;; 10 Turing Center for Living Systems, Aix-Marseille University, Marseille, France;; 11 Flathead Lake Biological Station, University of Montana, Polson, Montana, USA;; 12 Water Resources Science, University of Minnesota, St. Paul, Minnesota, USA;; 13 School of the Environment and Sustainability, University of Michigan, Ann Arbor, Michigan, USA;; 14 Department of Aquatic Sciences and Assessment, Swedish University of Agricultural Sciences, Uppsala, Sweden;; 15 Institute of Environmental Studies, Charles University, Prague, Czech Republic;; 16 School of the Environment, Washington State University, Richland, Washington, USA;; 17 Department of Natural Science, Hawai‘i Pacific University, Honolulu, Hawaii, USA;; 18 School of Natural and Environmental Sciences, Newcastle University, Newcastle upon Tyne, UK;; 19 Earth and Environmental Science, CUNY Advanced Science Research Center, Brooklyn College, New York, New York, USA;; 20 Department of Earth and Environmental Sciences, City University of New York Advanced Science Research Center at the Graduate Center and Brooklyn College, New York, New York, USA;; 21 Department of Biology, Baylor University, Waco, Texas, USA

## Abstract

Biological nitrogen fixation is the conversion of dinitrogen (N_2_) gas into bioavailable nitrogen by microorganisms with consequences for primary production, ecosystem function, and global climate. Here we present a compiled dataset of 4793 nitrogen fixation (N_2_-fixation) rates measured in the water column and benthos of inland and coastal systems via the acetylene reduction assay, ^15^N_2_ labeling, or N_2_/Ar technique. While the data are distributed across seven continents, most observations (88%) are from the northern hemisphere. ^15^N_2_ labeling accounted for 67% of water column measurements, while the acetylene reduction assay accounted for 81% of benthic N_2_-fixation observations. Dataset median area-, volume-, and mass-normalized N_2_-fixation rates are 7.1 *μ*mol N_2_-N m^−2^ h^−1^, 2.3 × 10^−4^ *μ*mol N_2_-N L^−1^ h^−1^, and 4.8 × 10^−4^ *μ*mol N_2_-N g^−1^ h^−1^, respectively. This dataset will facilitate future efforts to study and scale N_2_-fixation contributions across inland and coastal aquatic environments.

## Background and motivation

Nitrogen fixation (N_2_-fixation) or diazotrophy is a critical process in the nitrogen cycle that converts the largely unreactive pool of dinitrogen (N_2_) gas to biologically available ammonium. N_2_-fixation can relieve nitrogen limitation, enhance primary production, and ultimately alter global climate (Falkowski, Fenchel, and Delong 2008). There have been widespread research efforts to synthesize N_2_-fixation rate measurements in terrestrial and open ocean ecosystems (e.g., Cleveland et al. 1999; Luo et al. 2012; Vitousek et al. 2013; Shao et al. 2023). However, N_2_-fixation also occurs across inland and coastal waters (defined here as lakes, rivers, freshwater wetlands, salt marshes, mangroves, tidal flats, estuaries, and continental shelves). Despite a steadily growing body of work that suggests N_2_-fixation occurs widely in these systems (Marcarelli, Fulweiler, and Scott 2022), there have been no recent global compilations of N_2_-fixation from these environments.

The last compilation of N_2_-fixation rates from inland and coastal waters was the foundational paper, published in *Limnology and Oceanography*, by Howarth et al. (1988a). Since then, numerous studies have measured N_2_-fixation primarily using the acetylene reduction assay (ARA), ^15^N_2_ labeling techniques, and, most recently, the N_2_/Ar technique. Additional studies have used environmental genomic techniques to identify the N_2_-fixing community and quantify their potential for N_2_-fixation in these ecosystems (e.g., Fernandez et al. 2020; Jabir et al. 2021; Hallstrøm et al. 2022). Despite these efforts, a general conceptual model on the biogeochemical significance or the ecological role of N_2_-fixation in inland and coastal waters has not emerged. For example, the relative importance of N_2_-fixation in balancing nutrient availability with phosphorus, trace metals, and other growth limiting resources remains widely debated (Howarth, Marino, and Cole 1988b; van Gerven et al. 2019; Marcarelli, Fulweiler, and Scott 2022). Further, there has been no consensus on the contributions of N_2_-fixation in the benthos of diverse aquatic ecosystems, nor have there been recent attempts to quantify the role inland and coastal aquatic system N_2_-fixation might play in the global nitrogen cycle (Fulweiler 2023). Inspired by the last few decades of work on N_2_-fixation across inland and coastal waters and a desire to better understand how it fits into ecological and biogeochemical processes from local to global scales, we undertook a systematic review of the literature.

In this data article, we present a dataset of 4793 N_2_-fixation rate measurements collected from inland and coastal aquatic systems. Each rate is accompanied by its geographic location, measurement technique, and temporal information (e.g., sampling date, season, etc.). Additionally, we include ancillary data that might be useful in explaining drivers of N_2_-fixation in the future, though these data were limited in availability. This is the most comprehensive nitrogen fixation dataset available for inland and coastal waters. It was built by the members of the first working group convened by the Aquatic Nitrogen Fixation Research Coordination Network (RCN), whose first objective was to synthesize the current state of knowledge on rates and biodiversity of N_2_-fixation for ecosystems along the freshwater-marine continuum (Marcarelli, Fulweiler, and Scott 2022, 2023). We present this extensive N_2_-fixation dataset as a tool for the global aquatic research community with the anticipation that it will inform future N_2_-fixation studies, from modeling exercises to experiments and new field observations. Additionally, we anticipate that this is the first version of this dataset, and it will continue to be updated (e.g., similar to Shao et al. 2023 for open ocean water columns) as more N_2_-fixation rates become available.

### Dataset description

This dataset is a compilation of measured N_2_-fixation rates from published literature that will facilitate data use and downstream analysis. The published data package has four files consisting of (1) references from which data were extracted, (2) N_2_-fixation rates, (3) ancillary data associated with rate measurements, and (4) a data dictionary describing all variables found in the three data files. Each reference and rate were assigned a unique identifier that is shared between data files.

1. The reference file (aquatic_N2fix_references.csv) contains identifying details for each source from which data were extracted, including authors, DOI, journal, title, and the location in the publication from which the data were extracted (i.e., table or figure numbers, supplemental, etc.). If data were extracted from a published dataset associated with a paper, the DOI of the dataset is also listed.
2. The N_2_-fixation rate file (aquatic_N2fix_rates.csv) contains the N_2_-fixation rates extracted from each reference and information related to the rate measurement, such as method used, incubation time (if reported), the date and geographic coordinates of the rate measurement, habitat, and substrate used to measure the rate.
3. An ancillary data file (aquatic_N2fix_ancillary.csv) contains additional variables that were measured alongside N_2_-fixation rates, as available (Table 3).
4. A data dictionary (data_dictionary.csv) is provided with variable descriptions and units for each column, including the file that contains each variable.

All data can be accessed via: https://doi.org/10.6073/pasta/333f651ca721da657d5fd0c393d26cf8. This link will direct users to the Environmental Data Initiative repository where all versions of the dataset can be accessed. As of this writing, the current version is v2.

### Dataset summary

**Fig. 1.**
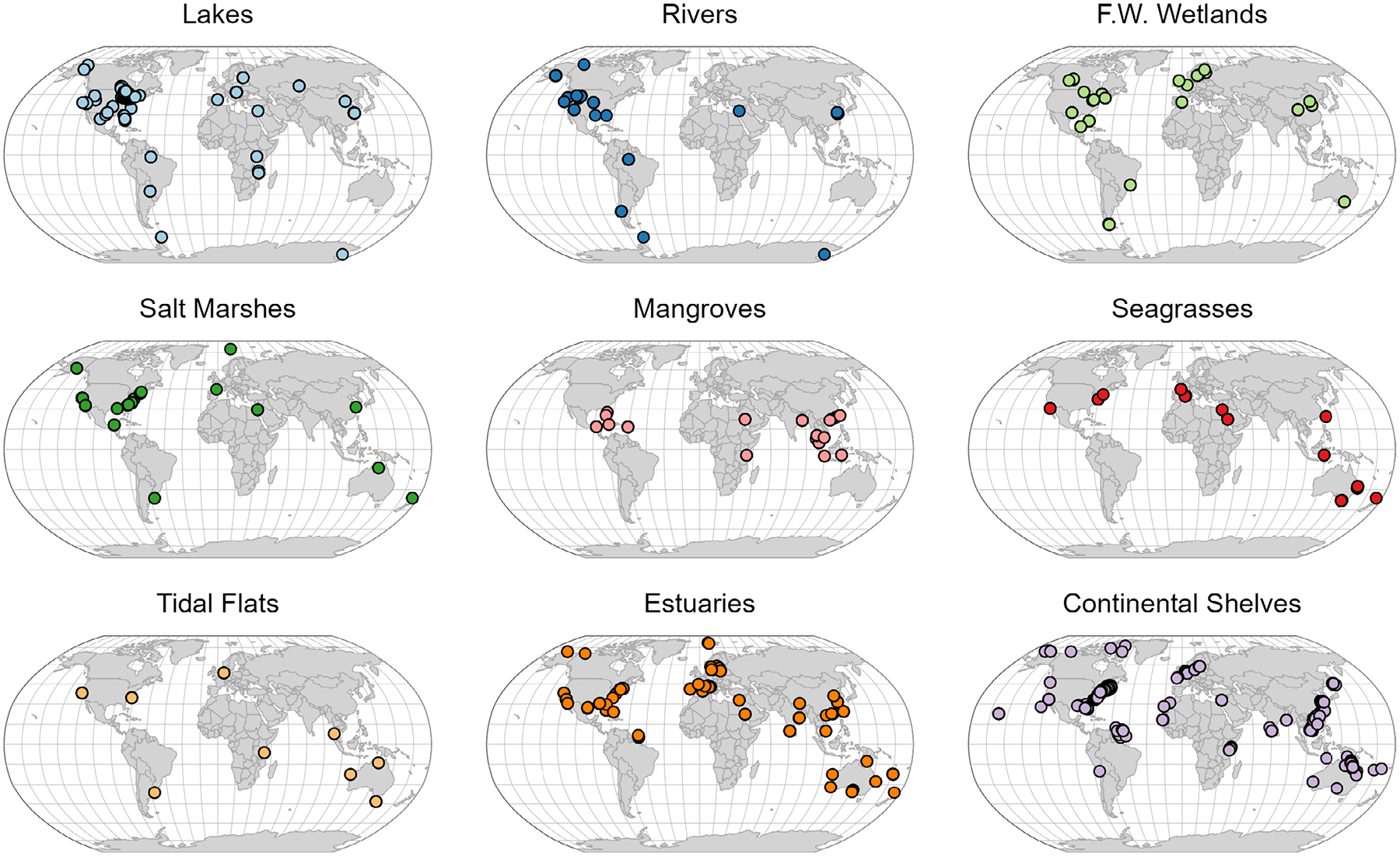
Geographic locations of N_2_-fixation rate measurements by habitat included in this dataset. F.W. Wetlands stands for freshwater wetlands.

**Fig. 2.**
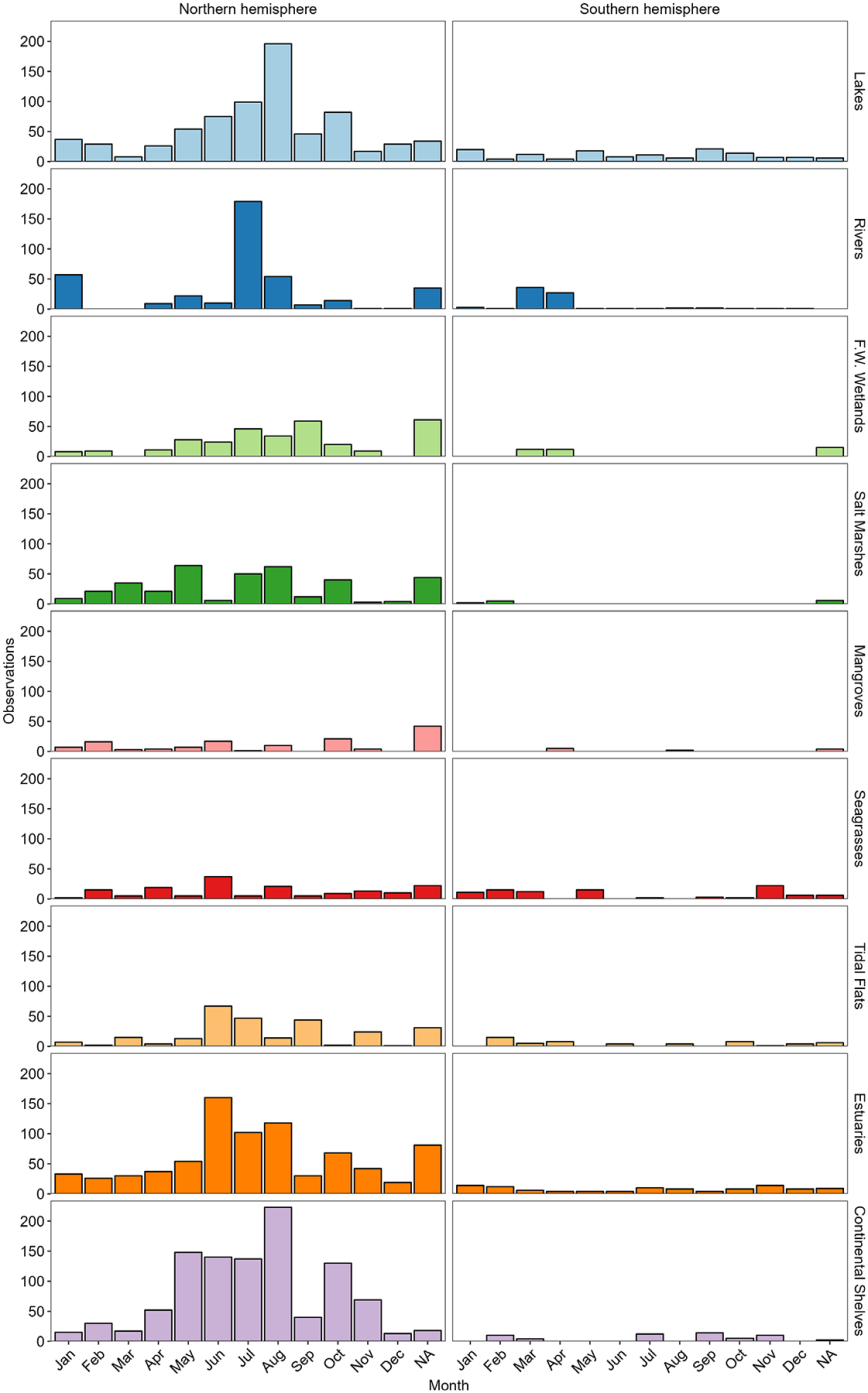
N_2_-fixation rate observations separated by reported sampling month for the northern and southern hemispheres (columns) and habitats (rows). NA means study did not report sampling month.

This dataset includes 4793 observations of N_2_-fixation rates across 267 data sources published before April 18, 2022, with between 1 and 225 individual observations reported per data source. N_2_-fixation rate measurements were distributed across all seven continents (Fig. 1), though mid-latitude northern hemisphere sites account for over 87% of reported rates (Fig. 2). Over half of the N_2_-fixation rates were from continental shelves (*n* = 1089, 22.7%), estuaries (*n* = 905, 18.9%), and lakes (*n* = 870, 18.2%), with fewer rates reported in rivers (*n* = 466, 9.7%), salt marshes (*n* = 384, 8%), freshwater wetlands (*n* = 348, 7.3%), tidal flats (*n* = 326, 6.8%), seagrasses (*n* = 262, 5.5%), and mangroves (*n* = 143, 3.0%) (Fig. 1). Reported N_2_-fixation rates are from all months of the year, though most are from May to October (*n* = 3153, 66%), which coincide with warm months and the active growing season of the northern hemisphere (Fig. 2).

We grouped rate measurements into three substrate categories: water column, benthos, and “other.” We defined benthos as anything related to the habitat bottom which included sediment, cyanobacterial or microbial mats, and intact plants incubated in sediment. In some cases, neither benthos nor water column was an accurate representation of the substrate and so we categorized these as “other.” The “other” category included rate measurements for dead plants or macrophytes (e.g., litter) or living vegetated parts such as leaves or roots. Benthic substrates were most frequently sampled (*n* = 2750, 57%), followed by water column (*n* = 1776, 37%) and “other” (*n* = 267, 6%).

N_2_-fixation rates were originally reported in over 60 different units (Table 1), which we converted to micromoles of N_2_-N per time and substrate area (*μ*mol N_2_-N m^−2^ h^−1^), substrate volume (*μ*mol N_2_-N L^−1^ h^−1^), or substrate mass (*μ*mol N_2_-N g^−1^ h^−1^) (*see* Methods section for details). We choose to report rates as N_2_-N (read N_2_ as N) because without specifying it can be unclear if reported rates are N_2_ or N, and because across scientific disciplines it is reported differently. Reporting it as N_2_-N is similar to how N concentrations can be reported for say nitrate concentrations as NO_3_-N, which is read as N in the nitrate molecule. N_2_-fixation rates range across several orders of magnitude both within and across habitats and substrates (Table 2). The median area-, volume-, and mass-normalized rates were 7.1 *μ*mol N_2_-N m^−2^ h^−1^, 2.3 × 10^−4^ *μ*mol N_2_-N L^−1^ h^−1^, and 4.8 × 10^−4^ *μ*mol N_2_-N g^−1^ h^−1^, respectively. We note that less than 7% of the extracted N_2_-fixation rates were reported as zero or below detection (which we reported as zero).

**Fig. 3.**
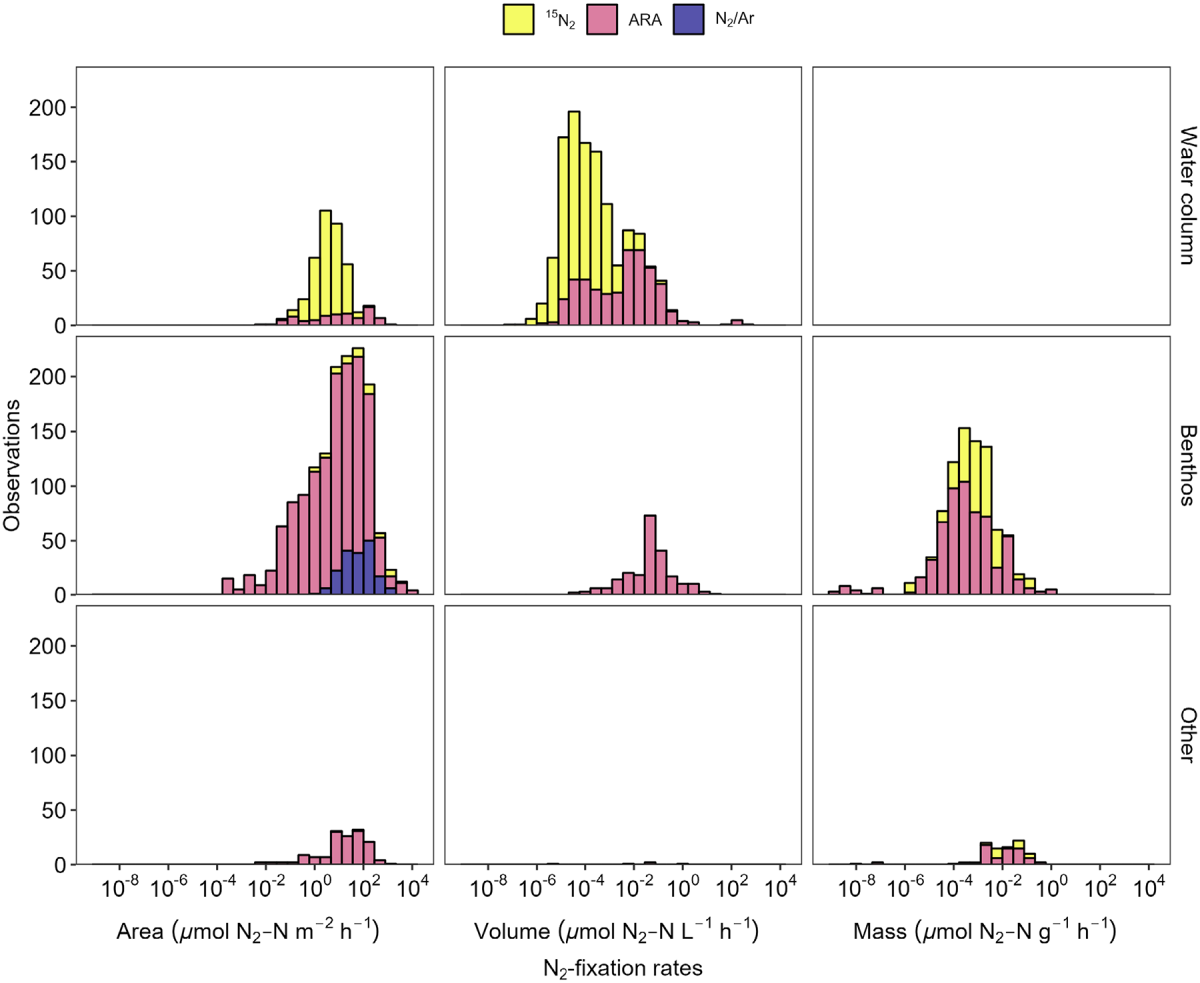
Histogram of N_2_-fixation rates by substrate (rows) and method (color).

**Fig. 4.**
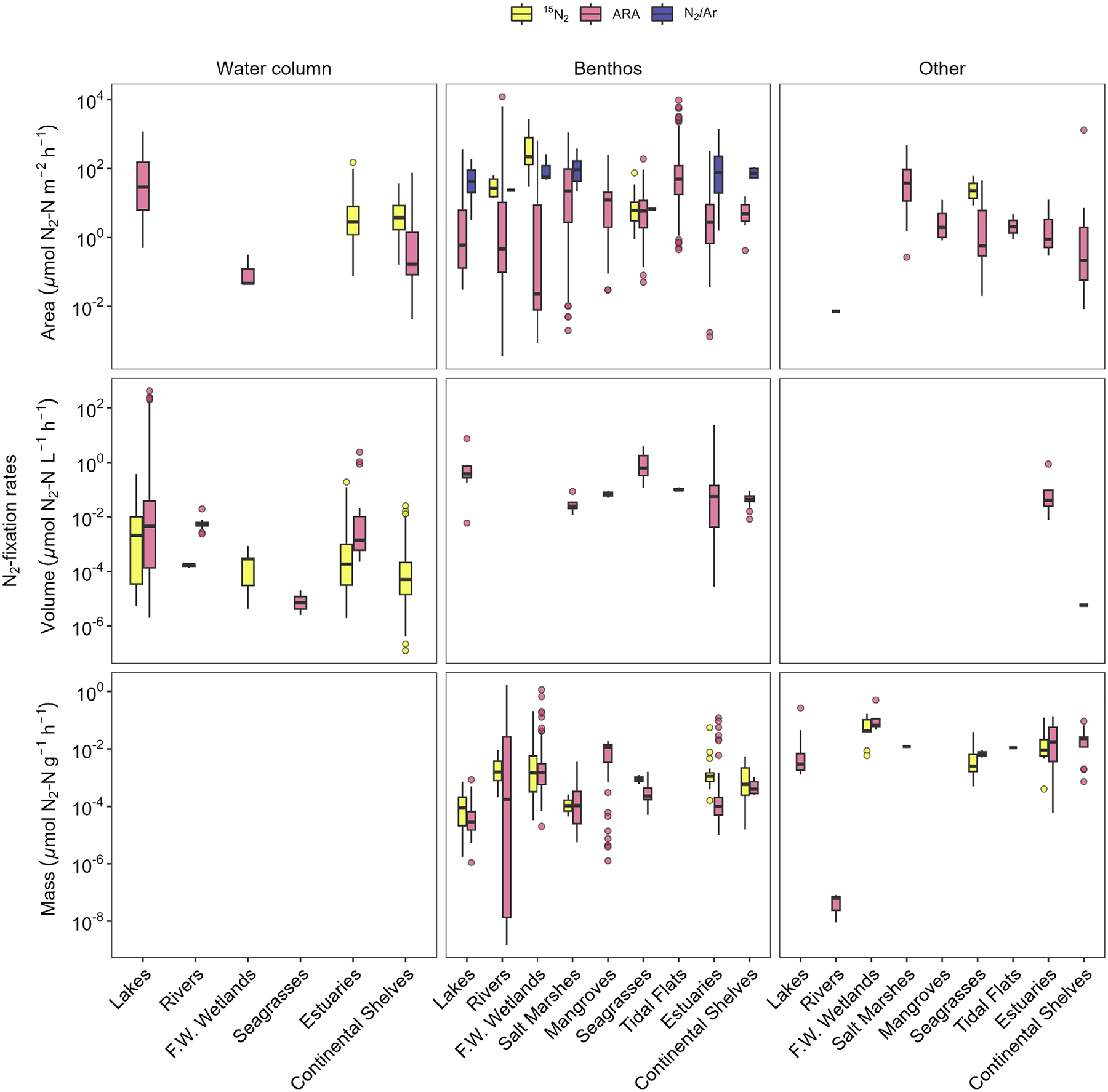
N_2_-fixation rates by habitat and method separated by units.

In this dataset, ARA was the most frequently used method for quantifying N_2_-fixation rates (64% of rates), followed by ^15^N_2_ tracer techniques (33% of rates). Of ARA rates that were converted into units of nitrogen by the data source authors (~ 64% of all ARA rates), a conversion factor of 3 mol ethylene produced per 1 mol of N_2_ fixed was used most frequently (65%), a factor of 4 to 1 was used 19% of the time, and the remaining 16% either did not report the factor used, used calibrated conversion factors, or used a different factor. The N_2_/Ar technique accounted for < 4% of rates, and this dataset included only benthic measurements (Fig. 3). Water column N_2_-fixation rates showed a distinct bimodal pattern with reported ^15^N_2_ fixation rates generally being lower than those measured by ARA (Fig. 3). For the benthos and other categories, N_2_-fixation rates followed similar patterns regardless of the method used; however, the magnitude of the rate varied substantially (Fig. 4). N_2_-fixation rate variability appears to be driven by habitat, substrate, and method (Fig. 4). The most pronounced example is for the benthos in lakes, freshwater wetlands, seagrasses, and estuaries, where median N_2_-fixation rates measured via ARA are 2–4 orders of magnitude lower than those measured by N_2_/Ar.

In addition to N_2_-fixation rates, we also extracted ancillary data that could be helpful in explaining variation in N_2_-fixation rates across studies and aquatic habitats. These included variables related to rate collection (e.g., incubation time or temperature) as well as environmental data (e.g., water column nutrient concentrations, sediment C : N). Unfortunately, most studies did not report or make such data readily available (Table 3). While most N_2_-fixation rates (*n* = 4345) could be paired to a reported duration of incubation, just over half reported the temperature of incubation, which is necessary for understanding measured rates relation to in situ conditions. Additionally, 65% of data were measured in brackish to saline habitats but salinity was only reported for 28% of those observations. Less than 20% of the N_2_-fixation rates also have concentrations of dissolved nitrogen and phosphorus, chlorophyll as a proxy for algal biomass, or dissolved oxygen—factors that are commonly considered drivers or constraints of N_2_-fixation (Table 3).

## Methods

### Literature search

**Fig. 5.**
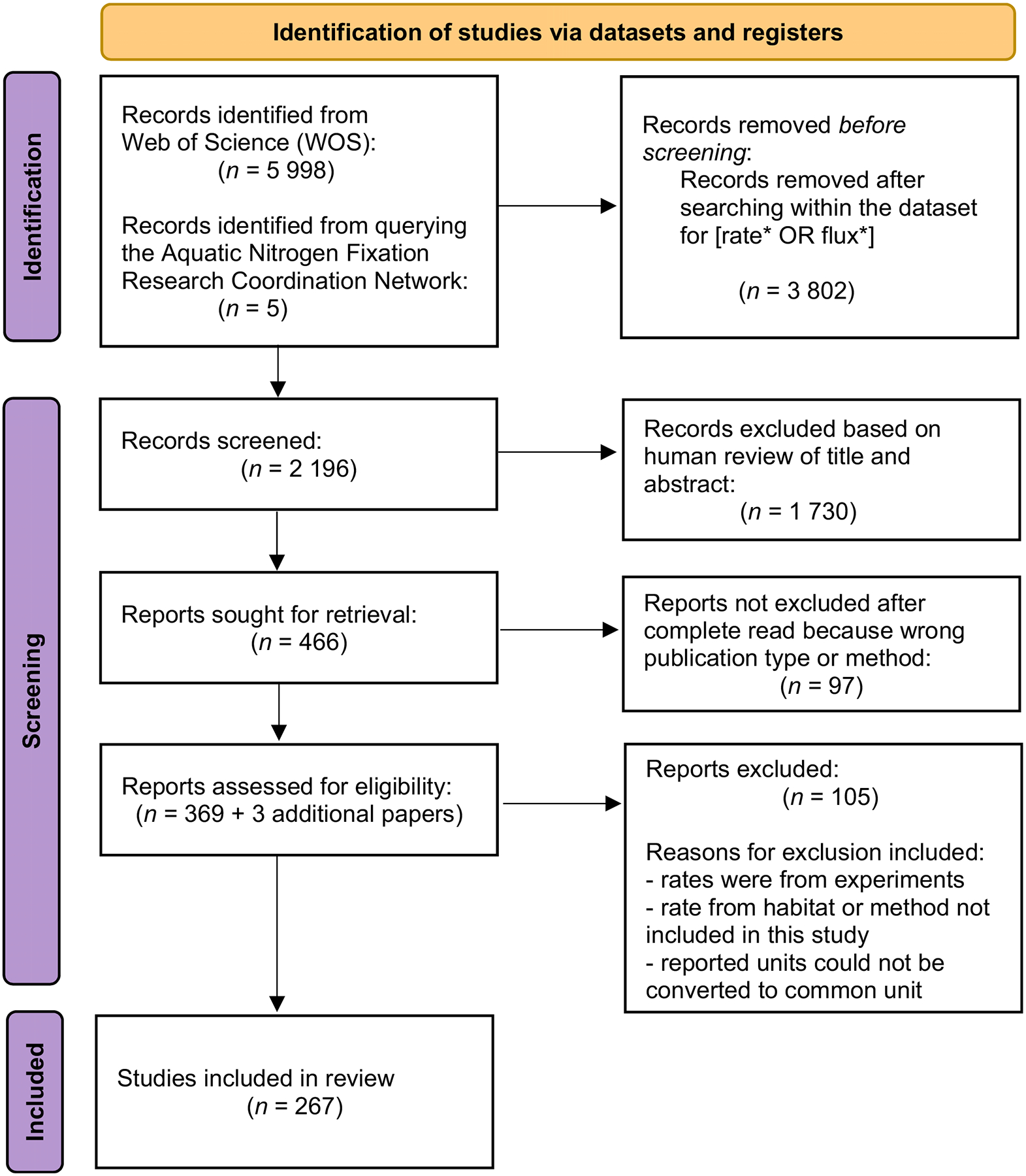
Literature selection flowchart for the development of this dataset.

We used the practices outlined by the Preferred Reporting Items for Systematic Reviews and Meta-Analyses to guide our systematic review (Fig. 5). Preferred Reporting Items for Systematic Reviews and Meta-Analyses was developed to provide standard methods and recommendations for conducting systematic reviews of health interventions and has been adapted for ecology and evolutionary biology studies (O’Dea et al. 2021). For this synthesis effort we primarily relied on the Preferred Reporting Items for Systematic Reviews and Meta-Analyses guidelines to develop and report our search string, as well as how we tracked which studies were included here.

To develop our search string, we used a modified version of the Population, Intervention, Comparison, Outcome approach (Foster and Jewell 2017). Specifically, we defined our population as the ecosystems found along the freshwater-marine continuum (e.g., lakes, streams, wetlands, estuaries, etc.), our variable was nitrogen, and our outcome was N_2_-fixation. This approach resulted in the following Boolean search string: (nitrogen OR (N2) OR dinitrogen) AND (fix*) AND (river* OR stream* OR ditch*) OR (lake* OR pond* OR reservoir* OR bayou*) OR ([aquaculture NEAR/0 pond*] OR aquaculture*) OR (wetland* OR swamp* OR bayou* OR fen* OR bog*) OR ((rice NEAR/0 padd*) OR (rice NEAR/0 agriculture*) OR (rice NEAR/0 wetland*)) OR (estuary* OR coastal OR (salt NEAR/0 pond*) OR (coastal NEAR/0 pond*) OR (coastal NEAR/0 lagoon*)) OR ((salt NEAR/0 marsh*) OR (tidal NEAR/0 marsh*) OR (coastal NEAR/0 wetland*) OR saltmarsh*) OR (mangrove* OR (mangrove NEAR/0 forest*)) OR (seagrass* OR (seagrass NEAR/0 bed*) OR (seagrass NEAR/0 meadow*)) OR ((tidal NEAR/0 flat*) OR (mud NEAR/0 flat*) OR (sand NEAR/0 flat*) OR mudflat* OR sandflat*) OR (shel* OR (continental NEAR/0 shel*) OR (coastal NEAR/0 shel*)).

We ran this search string through the Web of Science Core Collection (http://isiknowledge.com/) on April 17, 2022. This search identified 5998 records from Web of Science (Fig. 5). To refine our results further we searched within our results using the Boolean search string (rate* OR flux*), excluding review articles, book chapters, editorial materials, meeting abstracts, and retracted publications, which reduced the total number of records to 2196. These 2196 manuscripts were then screened for inclusion criteria.

### Criteria for inclusion

Studies were required to meet the following criteria to ensure reliability and usability:

1. *Rates were from peer-reviewed publications and were not part of a previous review*. All searches excluded review articles, book chapters, editorial materials, meeting abstracts, and retracted publications.
2. *Rates were from habitats within our scope*. We included rates from lakes and reservoirs, streams and rivers, freshwater wetlands, seagrass, mangrove and salt marsh habitats, tidal flats, estuaries, and continental shelves (up to 200 m). Rice farms were included in the freshwater wetland category. We excluded rates reported from coral reefs, aquaculture, and the open ocean.
3. *Geographic location was reported*. Observations were required to be associated with a specific geographic location. If geographic coordinates or a map with sampling locations were not included in the original publication, rates were not included in the dataset. In cases where a map was provided but coordinates were not reported, we used Google Earth to estimate latitude and longitude.
4. *N*_*2*_*-fixation rates were measured using a standard method*. We only included rates measured using the ARA, ^15^N_2_ tracer techniques, or net N_2_ flux using N_2_/Ar technique. We did not include positive N_2_ flux rates using ratios of N_2_/Ar, as these data suggest that net denitrification is occurring and provide no insights into the local magnitude of N_2_-fixation. We excluded rates that were derived from modeling or scaling approaches.
5. *Rates represented natural environmental conditions*. Rates from artificial substrates or culture studies were excluded. In cases of experimental manipulations, only the N_2_-fixation rates from control samples were included provided they represented natural, unaltered conditions.
6. *Rates were reported in scalable units*. Rates that were reported by area, volume, or mass of substrate were included. We removed rates that were reported in nonstandard units that required multiple assumptions to be made to convert to standard units (e.g., rates expressed in units of chlorophyll *a* concentration per time).

For screening, we exported the titles and abstracts from all 2196 manuscripts identified in our literature search into Rayyan (https://rayyan.ai) and removed any duplicates. Three co-authors led the review of these manuscripts. In Rayyan, all titles and abstracts were screened twice by two of three randomly assigned reviewers (Ouzzani et al. 2016) to identify papers that appeared to report measured rates of N_2_-fixation. Manuscripts that were identified as acceptable by both randomly assigned reviewers were retained. Manuscript titles and abstracts with split decisions were reviewed by the third reviewer and discussed by all three reviewers before final retention decisions were made. This initial screening reduced our analysis set to 466 manuscripts. These 466 manuscripts were then read in full by at least one reviewer to determine their eligibility for inclusion in our meta-analysis; this step identified 97 manuscripts that did not meet our criteria, reducing the number of manuscripts to 369 (Fig. 5). After these efforts, we also queried the N_2_-fixation community involved in the first Aquatic Nitrogen Fixation RCN working group to ensure that key papers reporting N_2_-fixation rates in the target habitats were not missed during the initial search. This resulted in five additional papers, of which three had data that were able to be extracted, resulting in 372 manuscripts in our final set of papers for data extraction. Of the final candidate papers (369 from Preferred Reporting Items for Systematic Reviews and Meta-Analyses process and 3 from internal “word of mouth”), rates from 267 were included in the final dataset (Fig. 5).

### Data extraction

For each of the final 267 manuscripts, we extracted all direct measurements of N_2_-fixation rates or N_2_-fluxes from ecosystems along the freshwater-marine continuum. Before extraction began, we developed a spreadsheet template that included the key information that must be available for the study to be included (*see* criteria for inclusion above). Specifically, alongside each N_2_-fixation rate or N_2_-flux observation, we also recorded (1) latitude and longitude; (2) habitat (e.g., estuary, continental shelf, lake); (3) measurement method (i.e., N_2_/Ar, ^15^N_2_, ARA); (4) substrate sampled (e.g., water column, sediment, microbial mat, litter); and (5) experimental approach (e.g., in situ, cores). The spreadsheet template also included columns for ancillary data that the RCN working group thought would be useful for our understanding of N_2_-fixation (e.g., incubation temperature, sample depth, salinity, water column nutrient concentrations, etc.). Ancillary data were extracted if they were readily available.

We extracted data from manuscript tables, text, figures (via Web Plot Digitizer; Rohatgi 2020), and supplemental materials, or directly downloaded manuscript data from the repository cited within the text. If data were not easily obtained from the published materials, we requested data directly from authors. We extracted data at the finest level possible: we extracted all the data points from a figure or values from a table where possible but could only extract mean values in some cases. As a result, there was variability in the granularity of the data extracted, which ranged from 1 to 225 discrete rates extracted per study.

### Data processing

Habitats were initially recorded as described in source manuscripts, before we reclassified them into nine categories following Rosentreter et al. (2021). We defined “lakes” (1) as surface water bodies surrounded by terrestrial habitats and grouped reservoirs within this category. This classification includes both freshwater and saline systems. “Rivers” (2) included streams and were defined as habitats with flowing freshwater within a defined channel. “Freshwater wetlands” (3) included all freshwater habitats with hydric soils and hydrophytes, such as peatlands, bogs, fens, and rice farms. “Seagrasses” (4), “mangroves” (5), and “salt marshes” (6) were defined based on the presence of dominant vegetation (e.g., macrophytes or mangrove trees). “Tidal flats” (7) included any intertidal habitat with bare sediment. “Estuaries” (8) included near-shore habitats and were defined as the brackish interface where surface freshwaters mix with the ocean. “Continental shelves” (9) were defined as the regions between near shore systems and continental slopes (≤ 200-m depth).

We converted N_2_-fixation rates, originally reported in over 60 different units (Table 1), to micromoles of N_2_-N per time and substrate mass (*μ*mol N_2_-N g^−1^ h^−1^), substrate area (*μ*mol N_2_-N m^−2^ h^−1^), or substrate volume (*μ*mol N_2_-N L^−1^ h^−1^). Whether rates were normalized to substrate mass, area, or volume depended on how the rates were originally reported. If rates were reported in units of ethylene rather than N_2_-N (~ 35% of ARA studies), we assumed a ratio of 3 : 1 for ethylene produced to N_2_ fixed (Hardy et al. 1968; Seitzinger and Garber 1987). The number of significant figures reported varied by data source, and for consistency we rounded all rates to three significant figures. Any rates that were originally recorded as “below detection” were reported as zero. Latitude and longitude that were recorded in units other than decimal degrees in their original publication were all converted to decimal degrees.

### Technical validation

Due to the nature of this dataset, which was compiled from published studies, we were unable to perform technical validation of methods and assess the quality of individual data points from the studies that comprise this synthesis. As such, the quality of the rate measurements relies upon quality checks performed by the authors and peer reviewers of the individual studies included here. Our data processing steps, and inclusion criteria were designed to remove studies that did not contain sufficient methodological, spatial, or temporal detail to be deemed reliable enough for inclusion in this dataset. Specifically, papers were required to report or display a map of geographical coordinates, and report habitat, measurement method, substrate used for the incubation, and experimental approach.

The remainder of the validation efforts focused on quality assurance for data extraction. Specifically, these data underwent a quality assurance check by at least one secondary reviewer prior to being included in the dataset. Any discrepancies or questions about the study-specific datasets were addressed by a team of co-authors participating in the first working group of the Aquatic Nitrogen Fixation RCN. Over the course of building this dataset and performing our quality assurance measures we estimate that 50% of the data sources were checked for rate extraction errors. In instances where errors were found, rates were manually re-extracted. To differentiate between rates from benthic substrates and the water column, a team of co-authors from the RCN working group designated substrate groups (water column, benthos, or “other”) for each study. These designations were verified by a different team of authors from the same working group. Similarly, all geographic coordinates were checked by a team of co-authors and corrected as needed. All coordinates were manually checked on Google Earth (Google Earth 2022) to verify their match with the described study location and re-extracted if necessary.

Variables contained in the ancillary data file did not undergo the same level of data extraction quality assurance as those found in the rates data file, which may result in an incomplete or inaccurate reflection of the data presented in the original sources. However, we have included the file because it provides a starting point for future data extraction efforts, and it demonstrates the need for more thorough ancillary data reporting in the future (*see* below).

### Data use and recommendations for reuse

Use of this dataset should cite this paper and the dataset (https://doi.org/10.6073/pasta/333f651ca721da657d5fd0c393d26cf8). Potential users of the dataset can find all pertinent information for reuse in the metadata and data files of the EDI data repository.

When re-using data, users of the dataset should be aware that habitat and substrate classification was conducted by our group based on the habitat and substrate listed in the publication, as described in detail in our data processes and quality assurance steps. Original data sources may have used a finer-scale habitat or substrate characterization in their study, which are reported alongside the general habitat and substrate categories that we designated. Additionally, this dataset may be biased toward nonzero rates for several reasons including a lack of reporting zeros or nondetects in published papers (e.g., Barto and Rillig 2012), zeros being encompassed in mean rates (i.e., if reported rate was a mean of multiple replicates or measurements that included zeros), and challenges with extracting zeros from figures (e.g., the absence of a bar in bar plots when a rate was zero could be interpreted as no rate measured). Finally, the data in the ancillary data file have undergone less quality assurance and we recommend that users conduct their own quality checks on these ancillary data prior to using them.

### Best practice suggestions for future N_2_-fixation rate studies

**Fig. 6.**
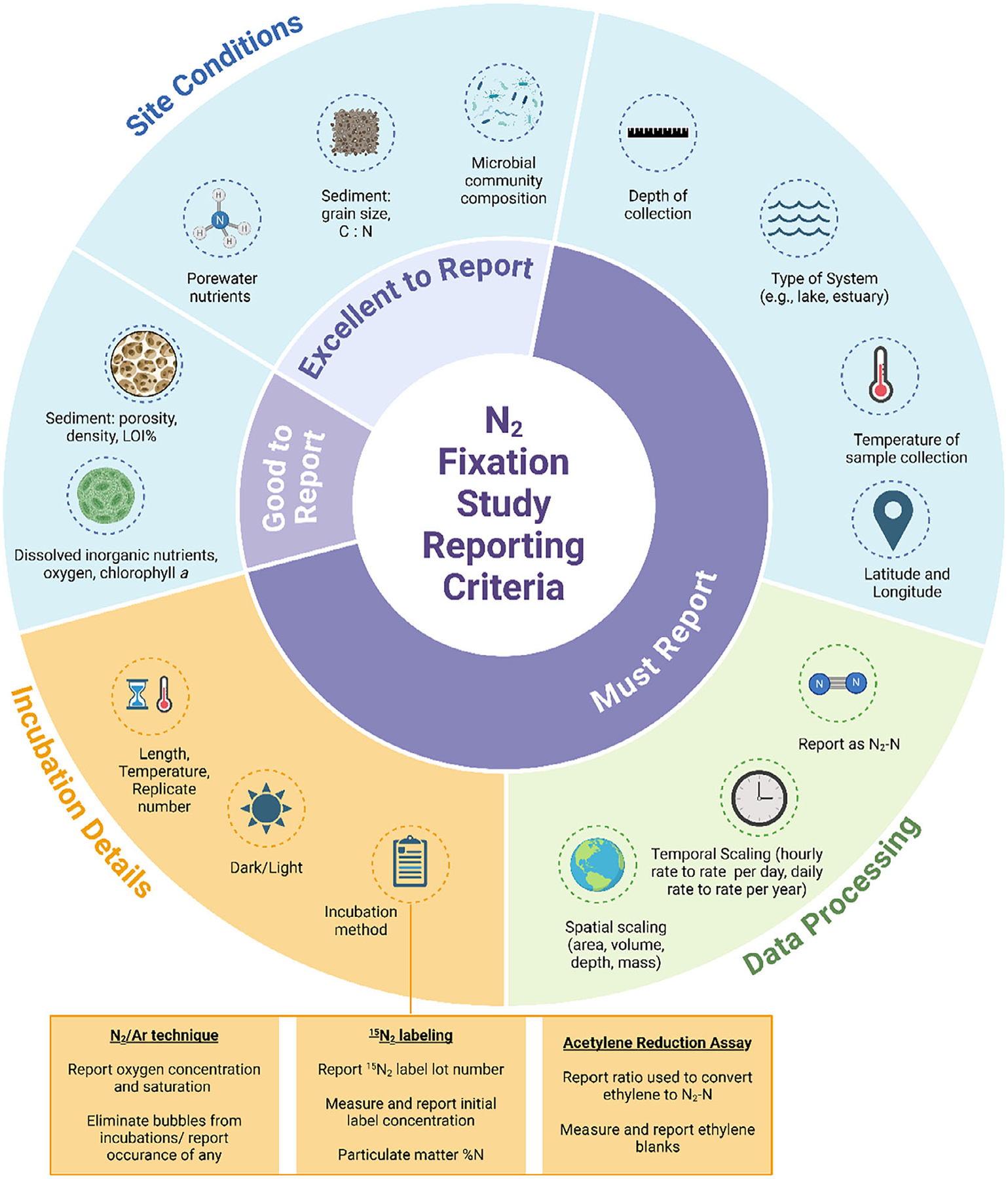
Overview of major recommendations to improve our understanding of N_2_-fixation across inland and coastal waters.

Through our work producing this dataset we have identified data reporting and methodological recommendations that will best advance inland and coastal water N_2_-fixation research (Fig. 6). We describe these ideas below in the hope that it motivates future work and provides guidance that will ease future synthesis efforts, and the continued expansion of this dataset.

#### Data availability

Fortunately, it is now standard practice for grants and journals to require that data are made publicly available alongside published manuscripts. Even when this is not the case, we urge our fellow N_2_-fixation enthusiasts to provide their data—both rates and ancillary environmental data—in easily accessible formats. We appreciate the recent movement toward open access datasets, whether required or voluntary, as data collection exercises like this one are labor intensive and difficult to sustain, nor are the resources available to do this for all processes or types of data. By being included in these syntheses, publicly available data have a continued and often far greater impact than the individual study that they were generated to support.

#### Site descriptions

It is important to provide enough background information to contextualize the ecosystem under study. Such information should include location (latitude and longitude) and a brief description of ecosystem type. Surprisingly, many studies do not report basic information on their study area, like whether the measurements were made in a lake or in an estuary, leaving it up to the readers to make their best guess. Additional descriptions should include depth and surface area of the system as well as any hydrological conditions such as stream discharge or tidal information.

#### Method considerations

##### ARA

The ARA is affordable and simple, which is reflected in its wide application in our dataset. However, there are limitations to the ARA method that make interpreting results more challenging. It is well known that acetylene can inhibit certain microbial groups including methanogens and sulfate reducers—two key functional groups that appear to drive N_2_-fixation in some habitats (e.g., sediments; Bertics et al. 2013, Fulweiler et al. 2013, Brown and Jenkins 2014, Jabir et al. 2021). Another consideration is that the 3 : 1 ethylene to N_2_ conversion appears to be more accurate for water column N_2_-fixation, compared to sediments where the conversion ratio can vary substantially (e.g., 10 : 1 to 100 : 1; Seitzinger and Garber 1987). Conversion ratios for each study should be determined and reported as conversion ratios may change depending on the type of sample being analyzed, the incubation temperature (Kunza and Hall Jr. 2023), as well as the presence of alternative nitrogenases (Soper, Simon, and Jauss 2021). Commercial acetylene can contain ethylene as can lab made acetylene from calcium carbide, although typically at a lower ethylene contamination level (Bytnerowicz et al. 2019). Ethylene is also produced and consumed by some microbes. Regardless of where it comes from any ethylene contamination makes it challenging to detect low N_2_-fixation, necessitating sufficient blanks and controls (*see* Bakker et al. 2022).

##### ^15^N_2_ labeling

The open ocean N_2_-fixation community has been actively working on improving water column N_2_-fixation methods for the ^15^N_2_ labeling method (e.g., Mohr et al. 2010; Dabundo et al. 2014; White et al. 2020). Many of the suggestions provided in those papers could benefit water column N_2_-fixation measurements for inland and coastal systems as well. Key takeaways from those efforts include measuring the concentration of particulate organic nitrogen as well as its ^15^N composition (i.e., time zero or natural abundance), measuring the amount of label added with isotope ratio mass spectrometry or membrane inlet mass spectrometry (i.e., not assuming label ^15^N concentrations based on gas dissolution calculations), reporting the lot number of ^15^N label (in case of contamination), reporting limits of detection, and included an appropriate estimate of error propagation. Conducting 24-h water column incubations is also helpful if reporting rates in days; however, shorter incubations can prove useful if interested in diel patterns of N_2_-fixation. Many of these suggestions would be welcome additions to benthic measurements as well.

##### N_2_/Ar

N_2_/Ar is an emerging methodology for measuring N_2_-fixation and as such there have been fewer method papers suggesting improvements for how to use it for N_2_-fixation rates. However, in general, when using the N_2_/Ar technique it is important to eliminate any bubbles, especially those made by oxygen (O_2_). This can be a particular challenge for water column measurements made in the light where O_2_ supersaturation conditions during photosynthesis can stimulate bubble formation which may preferentially diffuse N_2_ over Ar into bubbles, resulting in a loss of N_2_, that may be incorrectly attributed to N_2_-fixation. Similarly with sediments, in situ incubations or those done in the light risk the production of oxygen bubbles and subsequent misinterpretation of N_2_ loss as N_2_-fixation. One way to deal with this is to conduct dark incubations first, where oxygen will be drawn down well below saturation, followed by light incubations (Eyre and Ferguson 2005). Another option is to carefully inspect incubation containers for bubbles, although microbubbles might be hard to see, and to either remove or flag data as appropriate. One can also continuously monitor oxygen and not include N_2_ loss values if oxygen becomes supersaturated.

Open system approaches to using N_2_/Ar to estimate N_2_-flux including fixation is an emerging science that may help relieve some of these issues. For example, dissolved N_2_/Ar saturation ratios as either grab samples or diel signals have recently been used to estimate N_2_-N flux from lakes, streams, and wetlands (Reisinger et al. 2016; Loeks and Cotner 2020; Zhang, Li, and Pueppke 2022; Goeckner et al. 2024). Taylor et al. (2023) recently demonstrated good agreement between high rates of N_2_-fixation and undersaturated N_2_/Ar saturation ratios, but these measures are subject to multiple constraints including O_2_ supersaturation conditions mentioned above and accounting for broad variation in mixing and gas transfer dynamics across aquatic habitats. More research focused on constraining factors influencing relationships between N_2_/Ar saturation ratios and actual fluxes associated with N_2_-fixation is needed (Nifong et al. 2020).

#### Environmental characteristics

Because N_2_-fixation measurements are often collected in conjunction with other more routine measurements, we had initially hoped to examine environmental drivers of N_2_-fixation across inland and coastal waters. Unfortunately, we encountered a paucity of reported ancillary data in the papers that were screened for inclusion in this dataset. Information on temperature, pH, conductivity, and dissolved oxygen from water quality instruments or logging stations combined with any information on nutrient status (e.g., dissolved inorganic nitrogen, soluble reactive phosphorus, total nitrogen and phosphorus, dissolved organic or inorganic carbon) are supplemental measures that could help meta-analysis efforts describing global patterns in N_2_-fixation. Within benthic samples, characterization of sediments for grain size, carbon and nitrogen, or organic matter content is a potentially useful ancillary variable. Other important ancillary variables may include phytoplankton assemblage composition or sediment bacterial assemblage composition and activity. When collected, this information should be included in publications or datasets reporting N_2_-fixation to make data useful for larger scale efforts to identify ecological mechanisms driving N_2_-fixation across local, regional, continental, and global scales.

#### Units and scaling

One of the most challenging aspects of this synthesis was dealing with the wide range of units used to report N_2_-fixation (Table 1). While comparability between studies would be made easier if we consistently reported in an agreed upon set of units, we acknowledge this would be challenging given the subdisciplinary expectations and preferences that inform measurements across such a wide range of habitats. For example, oceanographers typically report rates in molar units, while freshwater scientists tend toward mass units. Wetland and benthic scientists and those interested in small-scale drivers tend to report rates per mass of substrate, while those whose motivation is to compare rates of different N cycling processes tend to report in units of surface area. Because we acknowledge this diversity in disciplinary preferences and research goals, we do not specifically recommend units here. At a minimum, we suggest that all N_2_-fixation rates should be reported as N_2_-N, which reads as N_2_ as N. This should be clearly stated in methods.

We do urge all researchers to provide all the spatial and temporal information needed and/or assumptions made for unit conversion (e.g., area sampled, sediment bulk density) to ensure that their data can be included in synthesis efforts such as this one. When reporting water column rates per unit area (m^2^), it would be helpful to include the depth over which the nitrogen fixation rate is integrated. When reporting water column rates per unit volume (e.g., L or m^3^), knowing the depth of measurements would allow for appropriate scaling to an area basis, which can then be more easily compared to areal estimates given for the benthos and terrestrial systems. When extrapolating N_2_-fixation measurements across time, it is necessary to include how hourly rates were converted to daily or annual rates. For example, some studies that report rates in units of per day assume that the rate of N_2_-fixation is continuous over 24 h, while others may only assume N_2_-fixation occurs during 10 or 12 daylight hours. Either assumption may be appropriate as heterocystous cyanobacteria fix N_2_ during daytime photosynthesis, while nonheterocystous taxa take advantage of elevated nighttime respiration to separate oxygen from nitrogenase (Inomura, Bragg, and Follows 2017). Stating these assumptions clearly and extrapolating results appropriately is critical for cross study comparisons. Similarly, it is necessary to know how daily rates are converted to annual rates. Some studies assume a 365-d N_2_-fixation year (which may be appropriate for a tropical system) while rates measured in a temperate region might be shorter, and polar shorter still. We note that the Howarth et al. (1988a) synthesis included detailed notes on how they scaled reported literature rates. While these detailed notes are helpful for understanding their work, the fact that our community still struggles with these same challenges decades later is a clear call to action. This dataset provides further evidence that all assumptions used in a study must be clearly stated.

#### Filling data gaps at the global scale

The current global N_2_-fixation dataset presented is biased toward mid latitude, northern hemisphere data points estimated from primarily continental shelf, estuary, or lake habitats and during spring and summer (i.e., the typical temperate system growing season). Refining our global understanding of N_2_-fixation across inland and coastal waters requires targeted efforts toward less represented habitats (e.g., rivers, freshwater wetlands, mangroves, tidal flats, and salt marshes) and less represented seasons (e.g., winter). There is also an urgent need for broader geographic coverage that includes more estimates from polar regions, subtropical and tropical regions, and greater coverage across the southern hemisphere. Finally, the comparability of different N_2_-fixation methods needs to be directly investigated, with the understanding that some methods may prove better suited for certain habitats.

### Comparison with existing datasets

To our knowledge, this is the most recent and comprehensive synthesis of N_2_-fixation rates from inland freshwater to coastal marine habitats, contributing toward filling an important gap in our understanding of global nitrogen cycling. Howarth et al. (1988a) estimated annual global N_2_-fixation rates from freshwater, estuarine, and marine systems with data available at the time, though the dataset described here is distinct from that effort in that it consists of compiled and formatted tabular data that are ready for downstream analysis, as well as 30+ yr of data. Capone and Carpenter (1982) published an estimate of marine benthic N_2_-fixation rates that is still widely used today to estimate the contribution of these systems to the global N budget (e.g., Voss et al. 2013). That budget did not include N_2_-fixation rates from tidal flats but did include rates for coral reefs which we do not include here. Comparable global N_2_-fixation synthesis efforts for terrestrial (e.g., Cleveland et al. 1999; Davies-Barnard and Friedlingstein 2020) and oceanic (Shao et al. 2023) systems have been published, though these do not include the aquatic systems that are our focus here. The notable lack of synthesis of N_2_-fixation rates across inland and coastal waters has limited our ability to account for these systems in global and regional budgets, and we anticipate this synthesis will spur renewed attention to biological nitrogen fixation in these systems.

## Figures and Tables

**Table 1.** Originally published N_2_-fixation units and number of references that report rates with that unit.

Mass units		Areal units		Volumetric units	
Original unit	References (n)	Original unit	References (n)	Original unit	References (n)
nmol-N g^−1^ h^−1^	16	*μ*mol-N m^−2^ h^−1^	38	nmol-N L^−1^ d^−1^	29
nmol-C_2_H_4_ g^−1^ h^−1^	8	mg-N m^−2^ d^−1^	18	nmol-N L^−1^ h^−1^	13
nmol-N_2_ g^−1^ h^−1^	8	*μ*mol-N m^−2^ d^−1^	17	nmol-C_2_H_4_ L^−1^ h^−1^	4
*μ*g-N g^−1^ d^−1^	5	*μ*mol-C_2_H_4_ m^−2^ h^−1^	16	*μ*g-N L^−1^ h^−1^	4
nmol-N_2_ g^−1^ d^−1^	3	nmol-C_2_H_4_ cm^−2^ h^−1^	12	ng-N L^−1^ h^−1^	3
*μ*mol-N g^−1^ d^−1^	3	mmol-N m^−2^ d^−1^	6	mg-N m^−3^ d^−1^	2
nmol-C_2_H_4_ g^−1^ d^−1^	2	*μ*g-N m^−2^ h^−1^	6	nmol-C_2_H_4_ cm^−3^ h^−1^	2
nmol-N g^−1^ d^−1^	2	mg-N m^−2^ h^−1^	5	nmol-C_2_H_4_ mL^−1^ h^−1^	2
*μ*g-N g^−1^ h^−1^	2	nmol-C_2_H_4_ cm^−2^ d^−1^	4	nmol-N cm^−3^ d^−1^	2
*μ*g-N kg^−1^ d^−1^	2	nmol-C_2_H_4_ m^−2^ h^−1^	4	nmol-N cm^−3^ h^−1^	2
*μ*mol-N g^−1^ h^−1^	2	*μ*mol-N_2_ m^−2^ h^−1^	4	*μ*g-N m^−3^ h^−1^	2
fmol-N g^−1^ h^−1^	1	g-N m^−2^ yr^−1^	2	*μ*mol-N_2_ L^−1^ d^−1^	2
ng-N g^−1^ d^−1^	1	mmol-N m-2 h^−1^	2	*μ*mol-N_2_ L^−1^ h^−1^	2
ng-N g^−1^ h^−1^	1	mmol-N_2_ m-2 d^−1^	2	mmol-C_2_H_4_ m^−3^ d^−1^	1
nmol-N kg^−1^ h^−1^	1	nmol-N cm^−2^ h^−1^	2	nmol-C_2_H_4_ cm^−3^ d^−1^	1
*μ*mol-C_2_H_4_ g^−1^ d^−1^	1	*μ*mol-N_2_ m^−2^ d^−1^	2	nmol-N m^−3^ h^−1^	1
*μ*mol-N kg^−1^ h^−1^	1	kg-N_2_ ha^−1^ yr^−1^	1	nmol-N_2_ cm^−3^ d^−1^	1
*μ*mol-N_2_ g^−1^ d^−1^	1	mg-N m^−2^ yr^−1^	1	nmol-N_2_ L^−1^ d^−1^	1
		mg-N_2_ m^−2^ h^−1^	1	nmol-N_2_ L^−1^ h^−1^	1
		ng-N m^−2^ h^−1^	1	*μ*g-N L^−1^ d^−1^	1
		nmol-C_2_H_4_ m^−2^ d^−1^	1	*μ*g-N L^−1^ h^−1^	1
		*μ*g-N cm^−2^ h^−1^	1	*μ*g-N_2_ m^−3^ d^−1^	1
		*μ*g-N_2_ m^−2^ h^−1^	1	*μ*mol-C_2_H_4_ L^−1^ d^−1^	1
				*μ*mol-C_2_H_4_ mL^−1^ 3 h^−1^	1
				*μ*mol-N L^−1^ d^−1^	1
				*μ*mol-N L^−1^ h^−1^	1

**Table 2.** Summary of median and interquartile range of N_2_-fixation rates by habitat, substrate, and reported units. The number of rates (*n*) reported in each unit is also shown.

		Area		Volume		Mass	
Habitat	Substrate	*μ*mol-N m^−2^ h^−1^	*n*	*μ*mol-N L^−1^ h^−1^	*n*	*μ*mol-N g^−1^ h^−1^	*n*
Lakes	Water column	17.4 (2.9–147)	72	0.00254 (5.92e-05–0.0296)	462	—	0
	Benthos	0.804 (0.156–15.4)	181	0.313 (0.003–0.546)	11	2.55e-05 (1.76e-06–8.82e-05)	129
	Other	—	0	—	0	0.00295 (0.00186–0.00872)	15
Rivers	Water column	—	0	0.00538 (0.0043–0.00612)	36	—	0
	Benthos	0.3 (0.00293–7.41)	269	0	1	0.00125 (0.000264–0.00464)	156
	Other	0.00714	1	—	0	6.33e-08 (3.63e-08–7.26e-08)	3
Freshwater wetlands	Water column	0.0464 (0.0464–0.179)	3	0.000146 (3.22e-06–0.000303)	8	—	0
	Benthos	37.3 (0.0158–176)	81	—	0	0.00147 (0.000472–0.0033)	242
	Other	—	0	—	0	0.0494 (0.0405–0.0941)	14
Salt marshes	Water column	—	0	—	0	—	0
	Benthos	22.7 (2.54–96.7)	225	0.0254 (0.0217–0.0411)	4	0.000107 (2.84e-05–0.000293)	45
	Other	37.9 (11.5–95.2)	109	—	0	0.0122	1
Mangroves	Water column	—	0	—	0	—	0
	Benthos	9.6 (0.533–17.6)	76	0.0722 (0.0614–0.0818)	3	0.0116 (0.00212–0.0141)	60
	Other	2.35 (1.01–5.82)	4	—	0	—	0
Seagrasses	Water column	—	0	1.13e-05 (6.93e-06–1.57e-05)	2	—	0
	Benthos	5.84 (1.79–11.7)	164	0.631 (0.343–1.79)	10	0.00024 (0.000178–0.000482)	60
	Other	0.57 (0.27–9.3)	19	—	0	0.00502 (0.00209–0.0078)	7
Tidal flats	Water column	—	0	—	0	—	0
	Benthos	45.6 (15.9–118)	320	0.0975 (0.0955–0.11)	3	—	0
	Other	2.83 (1.86–3.79)	2	—	0	0.011	1
Estuaries	Water column	2.79 (1.17–7.91)	113	0.000219 (2.76e-05–0.00113)	161	—	0
	Benthos	12.1 (2.22–96.3)	265	0.053 (0.00413–0.14)	138	0.00012 (5.39e-05–0.000365)	172
	Other	0.893 (0.596–6.7)	3	0.0415 (0.0298–0.254)	4	0.00583 (0.000298–0.0354)	49
Continental shelves	Water column	2.92 (0.875–6.9)	249	4.5e-05 (1.17e-05–0.000204)	669	0	1
	Benthos	2.92 (0–10.4)	27	0.0446 (0.0371–0.0592)	60	0.000575 (0.000258–0.00208)	48
	Other	0.217 (0.0583–1.97)	9	5.87e-06	1	0.00201 (0–0.0231)	25

**Table 3.** Percent coverage of select ancillary variables in this N_2_-fixation dataset. Ancillary variables included here are for only those with > 5% representation in the dataset. Variables that were required to be present for inclusion in the dataset (e.g., geographic coordinates, method, habitat) are not included.

File	Variable	Observation count	% of rates
Rate variables	N_2_-fixation incubation time	4345	90.7
	N_2_-fixation incubation temperature	2910	60.7
	Sample depth	2019	42.1
Ancillary variables	Salinity	1340	28.0
	Dissolved inorganic phosphorus (water column)	1036	21.6
	Ammonium (water column)	1006	21.0
	Chlorophyll *a* (water column)	1003	20.9
	Nitrate (water column)	860	17.9
	Dissolved oxygen (water column)	596	12.4
	Ammonium (sediment)	585	12.2
	pH	482	10.1
	Photosynthetic Active Radiation	478	10.0
	NOx (water column)	435	9.1
	C : N (sediment)	393	8.2
	Loss on Ignition (sediment)	383	8.0
	Dissolved inorganic nitrogen (water column)	358	7.5
	Nitrate (sediment)	320	6.7
	Iron (sediment)	293	6.1
	Chlorophyll *a* (sediment)	271	5.7
	Nitrite (water column)	260	5.4

## Data Availability

Dataset and metadata are available at https://doi.org/10.6073/pasta/333f651ca721da657d5fd0c393d26cf8 (Environmental Data Initiative, 2025).

## Scientific Significance Statement

Here we provide and describe a dataset of global nitrogen fixation rates for the water column and benthos of inland and coastal waters. This dataset is useful for understanding how nitrogen fixation varies across these ecosystems and is a resource for focusing future research questions. We anticipate this is the first version of this dataset that will continue to develop with newly published rates and expanded data on environmental drivers.
